# Development of Biodegradable Rigid Foams from Pineapple Field Waste

**DOI:** 10.3390/polym15132895

**Published:** 2023-06-29

**Authors:** Atitiya Namphonsane, Taweechai Amornsakchai, Chin Hua Chia, Kheng Lim Goh, Sombat Thanawan, Rungtiwa Wongsagonsup, Siwaporn Meejoo Smith

**Affiliations:** 1 Center of Sustainable Energy and Green Materials, Faculty of Science, Mahidol University, Phuttamonthon 4 Road, Salaya, Nakhon Pathom 73170, Thailandsiwaporn.smi@mahidol.ac.th (S.M.S.); 2 TEAnity Team Co., Ltd., 40/494 Soi Navamintra 111, Khet Bueng Kum, Bangkok 10230, Thailand; 3 Department of Applied Physics, Faculty of Science and Technology, Universiti Kebangsaan Malaysia, Bangi 43600, Selangor, Malaysia; chia@ukm.edu.my; 4 Mechanical Design and Manufacturing Engineering, Newcastle University in Singapore, 172A Ang Mo Kio Avenue 8 #05-01, SIT@NYP Building, Singapore 567739, Singapore; kheng-lim.goh@newcastle.ac.uk; 5 Faculty of Science, Agriculture & Engineering, Newcastle University, Newcastle upon Tyne NE1 7RU, UK; 6 Rubber Technology Research Center, Faculty of Science, Mahidol University, Phuttamonthon 4 Road, Salaya, Nakhon Pathom 73170, Thailand; sombat.tha@mahidol.ac.th; 7 Food and Nutrition Academic and Research Cluster, Institute of Nutrition, Mahidol University, Phuttamonthon 4 Road, Nakhon Pathom 73170, Thailand; rungtiwa.won@mahidol.ac.th

**Keywords:** biodegradable plastic, starch, circular economy, pineapple, tensile strength

## Abstract

Pineapple materials sourced from agricultural waste have been employed to process novel bio-degradable rigid composite foams. The matrix for the foam consisted of starch extracted from pineapple stem, known for its high amylose content, while the filler comprised non-fibrous cellulosic materials sourced from pineapple leaf. In contrast to traditional methods that involve preparing a batter, this study adopted a unique approach where the starch gel containing glycerol were first formed using a household microwave oven, followed by blending the filler into the gel using a two-roll mill. The resulting mixture was then foamed at 160 °C using a compression molding machine. The foams displayed densities ranging from 0.43–0.51 g/cm^3^ and exhibited a highly amorphous structure. Notably, the foams demonstrated an equilibrium moisture content of approximately 8–10% and the ability to absorb 150–200% of their own weight without disintegration. Flexural strengths ranged from 1.5–4.5 MPa, varying with the filler and glycerol contents. Biodegradability tests using a soil burial method revealed complete disintegration of the foam into particles measuring 1 mm or smaller within 15 days. Moreover, to showcase practical applications, an environmentally friendly single-use foam tray was fabricated. This novel method, involving gel formation followed by filler blending, sets it apart from previous works. The findings highlight the potential of pineapple waste materials for producing sustainable bio-degradable foams with desirable properties and contribute to the field of sustainable materials.

## 1. Introduction

Foam is a material that is characterized by a porous structure that contains gas-filled pockets. Such characteristics have brought numerous advantages to the material. They are lightweight, strong, and offer excellent insulation properties, making them ideal for a wide range of applications, from packaging and cushioning materials to building insulation and protective gear. Foam materials, such as polystyrene foam, have become ubiquitous in modern life due to the aforementioned advantages. However, their widespread use has also led to a range of environmental problems, including the accumulation of microplastics in the environment and the persistence of foam debris in marine ecosystems [1,2,3]. These issues have prompted many countries [4] and local governments [5,6,7] to ban or restrict the use of certain types of foam materials, highlighting the urgent need for more sustainable alternatives.

Because of the many advantages of foam structures, there has been a number of attempts to find alternative biodegradable materials for the production of foams. This has gained much attention in recent years due to its potential to reduce the environmental impact of foam production and disposal. Biodegradable foams can be made from a variety of materials, including plant-based sources such as starch [8,9,10,11], starch-based mixture [12,13,14,15,16] and soy-protein-based materials [17], as well as synthetic biodegradable polymers [18,19,20,21,22]. While these materials may seem to offer many advantages, including reduced toxicity and biodegradability, they are certainly not easily biodegradable and require an industrial composting facility [23,24,25]. They can also require significant amounts of energy to produce and may compete with food crops for resources, raising concerns about their overall sustainability.

To address these issues, researchers are exploring different routes such as mycelium composites [26,27,28], foam [29,30] and leather [31,32]. We are exploring the use of agricultural waste as a source of raw material for foam production. Pineapple is one of the most widely produced tropical fruits in the world, and its leaves, stems, and fruit residues generate significant amounts of waste. Pineapple field waste contains cellulosic material [33,34,35,36] and starch [37,38,39] which can be used to produce more sustainable and biodegradable foam materials. By converting this waste into biodegradable foam, we could not only reduce waste and its environmental impact [40,41] but also promote circular bioeconomy, where waste is used as a resource.

The specific objectives of this study are to develop a biodegradable foam using pineapple field waste as raw materials and to assess its suitability as a sustainable alternative to synthetic foam. Our aims include evaluating the mechanical properties, internal structure, water sensitivity, and the biodegradability of the composite foams produced using pineapple stem starch as the matrix, pineapple leaf material as filler, glycerol as a plasticizer, and water as a blowing agent. Additionally, we aim to showcase the material’s potential applications through the fabrication of a packaging tray.

## 2. Materials and Methods

### 2.1. Raw Material and Chemicals

Pineapple stem waste was from Hong Mao Biochemicals Co., Ltd. (Rayong, Thailand). It was a byproduct of a proprietary bromelain extraction process in which, according to general principle, peeled pineapple stems were crushed to disrupt the cell structure and liquid extracted by centrifugation [42]. The remaining solid material was dried under the sun for a few days and further ground into powder using a laboratory grinder. The stem powder was collected by sieving (80 mesh) to separate the coarse fibers, cell wall, and other solid contaminants, which constitute about 56% of the whole mass. The powder obtained has similar characteristics to that obtained by the wet milling reported previously [35]. The non-fibrous component of pineapple leaf waste was obtained by grinding fresh pineapple leaves according to the method previously described in [29,30]. The ground material was dried and then sieved with plastic wire mesh of approximately 2 mm^2^ to separate out the puffy fibrous component. Only the particulate non-fibrous material (NFM) that passed through the sieve was used as filler. Glycerol was commercial grade and obtained from local stores.

### 2.2. Preparation of Starch Paste and Composite Foams

Starch paste was prepared by mixing a predetermined amount of PSS, water, and glycerol in a glass beaker. The amount of glycerol was varied at 10, 15, and 20% and NFM at 15, 20, 25, and 30% based on the weight of PSS (Table 1). For all formulations, the amount of water was fixed at the same weight as PSS. The formulations and codes are shown in Table 1. PSS, water, and glycerol were mixed in a beaker and the mixture was left for at least 60 min at ambient conditions before being gelatinized in a household microwave (Toshiba, model ER-G33SC(S), Toshiba Thailand Co., Ltd., Nonthaburi, Thailand) set at 50% of maximum power (1100 W) for a total time of 80 s. The mixture was taken out to mix with a glass stirring rod every 10 s. The gelatinized PSS was covered to prevent moisture loss and left to cool down to room temperature before being further processed. A predetermined amount of NFM (15, 20, 25, and 30% weight of PSS) was then added into the paste on a laboratory 2-roll mill and mixing continued until a homogeneous mixture was obtained. The mixing time was approximately 15 min. After mixing, the mixture was sheeted out to a thickness of approximately 2.0 mm. The sheet was then brought to foam in a compression molding machine. The mixture was cut into a square shape of size 150 mm × 150 mm and placed between two flat steel sheets with a 3.5 mm thick spacer. The whole mold was put in a compression molding machine set at 160 °C for 5 min and then cooling under pressure for another 5 min. The foam samples were then left in ambient environment to gain equilibrium moisture content at least 7 days before any measurement was made.

### 2.3. Characterization of PSS Composite Foams

#### 2.3.1. Foam Density

Apparent density of the foam was determined by dividing the mass of a square specimen by its apparent dimension. A square piece of sample with dimension approximately 20 × 20 mm^2^ was cut from a compression molded sheet of thickness 3.5 mm. The piece was then weighed to 4 decimal places with a laboratory balance (XS105, Mettler Toledo, Greifensee, Switzerland). The density was then calculated from mass divided by volume (20 × 20 × thickness mm^3^). An average value from five specimens was reported.

#### 2.3.2. X-ray Diffraction (XRD)

X-ray diffraction patterns of the materials were obtained from a benchtop X-ray powder diffractometer (D2 Phaser, Bruker AXS GmbH., Karlsruhe, Germany) using X-ray wavelength of 1.54 Å with a step scan of 15 s/point over the 2θ of 5–40 degrees.

#### 2.3.3. Mechanical Properties

Flexural test: Flexural properties of the composite foams were determined on a universal testing machine (Instron 5569, Instron, High Wycombe, UK) at a crosshead speed of 1 mm/min, 5 kN of load cell with the support span length of 56 mm. The specimens were cut into strips that were 11.4 mm wide. Flexural strength (*σ*) and modulus (*E*) were determined from Equations (2) and (3), respectively. The values reported were average values from five specimens.

(1) $$\sigma =\frac{3FL}{2bh^{2}}$$

(2) $$E=\frac{L^{3}F}{4bh^{3}d}$$

where

*F* is force (N) at peak position (for *σ*) and 1% deflection (for *E*);
*L* = width of the beam (mm);
*b* is the width of specimen;
*h* is the height of the samples; and
*d* = deflection (mm).

The averaged values of flexural strength and flexural modulus from five specimens were reported.

#### 2.3.4. Morphology

Fractured surfaces of the foams were observed with a scanning electron microscope (SEM) (JSM-IT500, JEOL, Tokyo, Japan). The samples were coated with platinum before the observation.

#### 2.3.5. Water Solubility, Water Absorption and Moisture Content

Water solubility: A piece of foam was cut from the compression molded sheet into the size of 20 × 20 mm^2^ and dried in an air ventilated oven set at 80 °C for 24 h. Its dried weight was determined (*w*_i_) before being immersed in distilled water with gentle stirring for 24 h. The sample was then dried at 80 °C for 24 h and its weight determined again (*w*_fd_). Water solubility was calculated from the following equation.

Water solubility (%) = ((*w*_i_ − *w*_fd_)/*w*_i_) × 100 (3)

Water absorption: The test was conducted in a similar manner to water solubility except that the sample was taken out at 5, 10, 20, 30, 60 and 120 min after immersing in distilled water. Excess water on the sample surface was removed with blotting paper and its weight determined (*w*_f_). Water absorption was calculated from the following equation.

Water absorption (%) = ((*w*_f_ − *w*_i_)/*w*_i_) × 100 (4)

Moisture content: A 20 × 20 mm^2^ piece of foam was left in laboratory ambient for at least 7 days to reach equilibrium and its weight determined (*w*_i_). After that, it was dried in an air-ventilated oven at 80 °C for 24 h. Its weight was determined (*w*_d_) again and the moisture content of the sample was calculated from the following equation.

Moisture content (%) = (*w*_i_ − *w*_d_)/*w*_d_ × 100 (5)

#### 2.3.6. Soil Burial Test

The biodegradability of starch-based materials by microorganisms [43] was evaluated using soil burial test. The test was slightly modified from a protocol reported previously [43]. Specimens of size 4.0 × 4.0 cm^2^ were cut and put in envelopes made from a high-density polyethylene net for easy recovery. The envelopes were buried in the edge of a garden of the department building about 10 cm beneath the surface. The pH of the soil was measured to be 7.5. The area was under the shade of trees and was watered every week. No attempt was made to regulate the moisture content and temperature of the area to obtain a natural environment. Envelopes were taken out for the observation of samples after different periods of time. The state of biodegradability was evaluated visually.

### 2.4. Statistical Analysis

Statistical analysis was performed using analysis of variance (ANOVA) with the Data Analysis tool in the Microsoft Excel (Office16) program. The *t*-test method, with two-sample assuming unequal variances, was performed to analyze differences among the means at a confidence level of 95%.

## 3. Results

### 3.1. Foam Appearance and Structure

**Figure 1:**
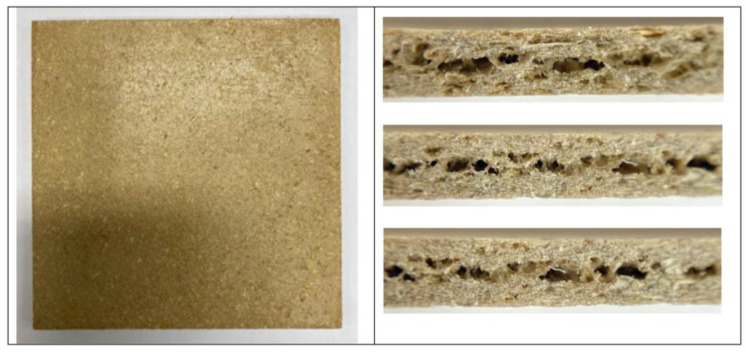
Photographs of PSS/NFM foams, depicting the top view (**left**) and the structure across the thickness and along the edge (**right**).

Figure 1 displays the visual image of rigid foam sheet prepared from PSS/NFM composite. The foam sheet has a light gold color with a smooth and dense surface with porous internal structure. Table 2 displays the densities of foams containing different amounts of glycerol and NFM. There appears to be an increase in densities with increasing NFM contents while no apparent change with glycerol content. The densities of PSS/NFM foams fall in the range observed in tapioca starch/PBS foam [22] but much greater than that of synthetic polymer foams such as expandable polystyrene (EPS) which ranges from 0.0031–3.5 g/cm^3^, with an average value of about 0.201 g/cm^3^ [44]. The densities of our PSS/NFM foams also fall in the range of other starch-based foams such as 0.20–0.60 g/cm^3^ [45,46,47,48]. It is worth noting that starch foam density has been found to depend on amylose content [11,48]. The higher the amylose content, the greater the density, from 0.082 for non-amylose-(0%) starch to 0.40 for high (70%)-amylose starch foam. In addition, foam density depends on foam technique, and baking usually provides foams with greater density than extrusion [11], presumably due to the much higher pressure differences in the extrusion process.

### 3.2. X-ray Diffraction (XRD)

**Figure 2:**
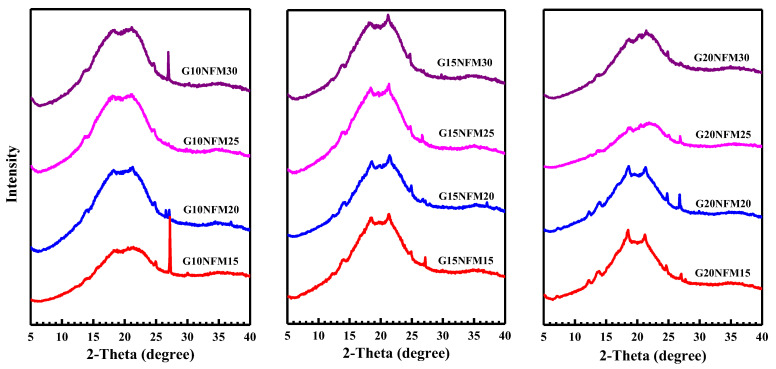
XRD of PSS/NFM foams.

Figure 2 displays XRD patterns of PSS composite foams containing different amounts of glycerol and NFM. All PSS composite foams are largely non-crystalline. Some composites, such as G15NFM15, G15NFM20, G15NFM25, G15NFM15 and G20NFM20, have limited crystallinity. This is very different from pineapple stem starch films that were obtained by solution casting [49,50] which exhibit much clearer and larger crystalline peaks. These crystalline peaks are attributed to the spontaneous recrystallization of amylose molecules during film drying [51,52] or retrogradation and the structure is always B-type regardless of the starch type [53,54]. This absence of crystallinity is due to the fast drying during compression molding; therefore, the amylose molecules did not have enough time to crystallize despite their high ability to undergo rapid reordering to form double helices and crystallites of amylose [55,56]. The XRD patterns in Figure 2 are similar to that of high-amylose (70%) baked corn-starch foam [10], i.e., highly amorphous with very small crystalline peaks at about 17° and 22°.

### 3.3. Internal Morphology

**Figure 3:**
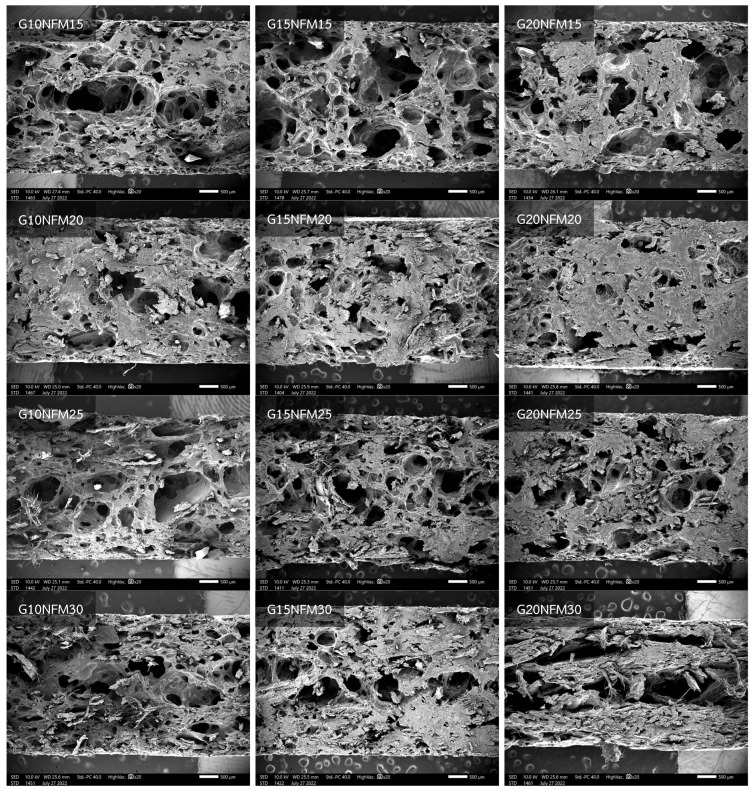
SEM of cross-section of PSS/NFM composite foams.

Figure 3 displays scanning electron photomicrographs of PSS/NFM composite foams with different formulations. All the foams exhibit an open-cell structure, evident not only from visual examination but also from other observations. For instance, during water absorption measurements, the foams displayed the characteristic behavior of sinking, indicating the presence of interconnected cells. Furthermore, compared to baked foams characterized by very large thin wall voids in the core region and a dense skin [11], these composite foams demonstrate relatively thick walls. This can be attributed to the presence of NFM particles, which thicken the starch paste and hinder significant expansion. When comparing foams with different formulations, limited deductions can be made, except that it appears the foam with the lowest glycerol content and lowest NFM content exhibits larger voids in the core region. As the glycerol and NFM content increases, void sizes seem to decrease and become more uniform.

### 3.4. Mechanical Properties

**Figure 4:**
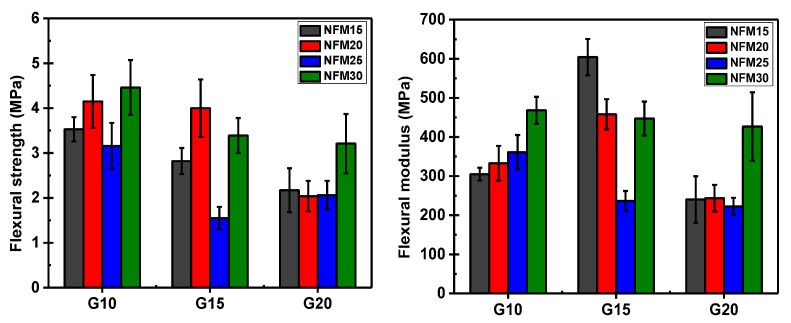
Flexural strength (**left**) and flexural modulus (**right**) of PSS/NFM foams.

Flexural strength and flexural modulus of PSS/NFM composite foams are shown in Figure 4. In general, flexural strength increases with increasing NFM content and decreases with increasing glycerol content. This is to be expected as NFM is the solid cellulosic material while glycerol plasticizes the system. For the flexural modulus, although similar behavior is to be expected, the results appear to be rather scattered. Only with a glycerol content of 10% (G10) that the mentioned pattern is discernible. For that, with glycerol content of 15% (G15), the lowest NFM content (G15NFM15) displays the highest modulus and the modulus decreases as the NFM increases. No exact explanation can be offered at present, but it could well be related to the internal structure of the foam which is known to strongly influence the flexural modulus. However, it is worth noting that the values of flexural strength and modulus are close to or even higher than most foam starches [11].

### 3.5. Moisture Content, Water Solubility and Absorption

**Figure 5:**
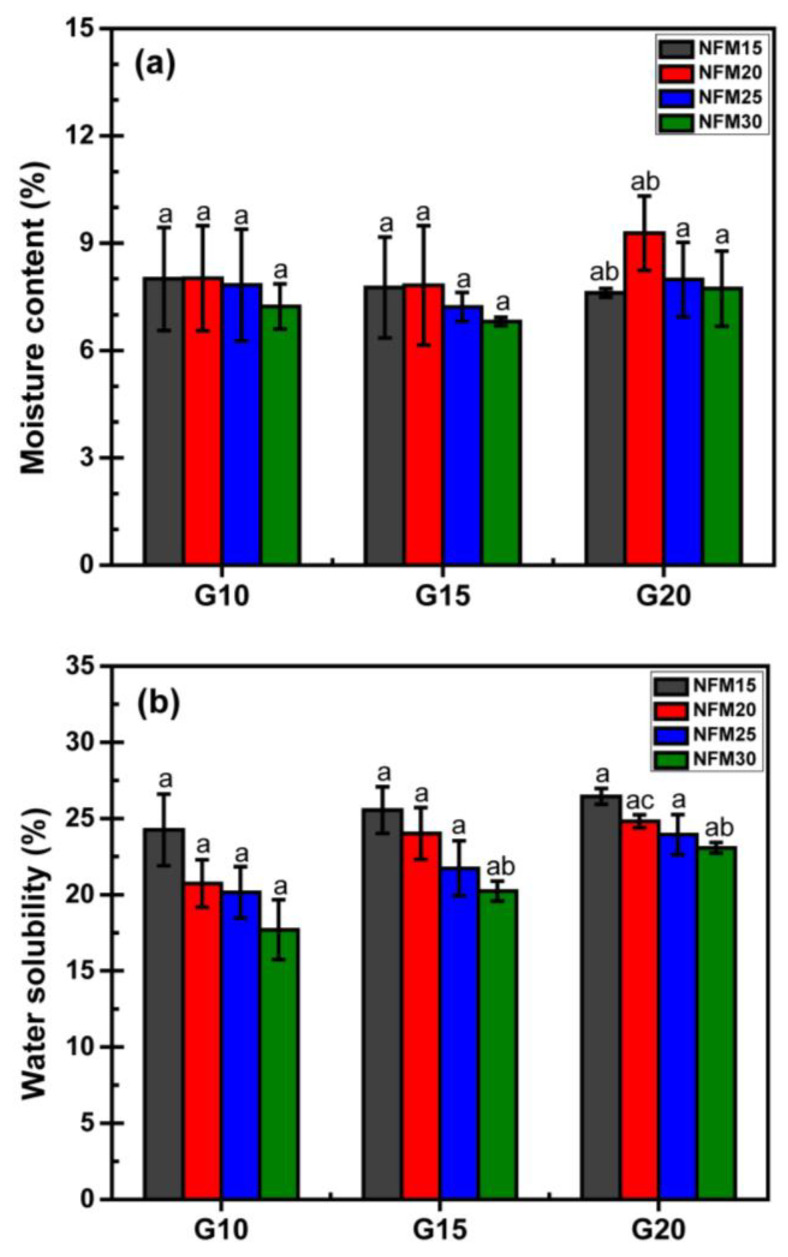
Moisture content (**a**), and water solubility (**b**) of PSS/NFM foams.

Figure 5a illustrates the equilibrium moisture content of PSS/NFM composite foams, with an average moisture content ranging between 7.0% and 9.5%. Despite the relatively large deviation, it can be concluded that the moisture content is not significantly influenced by the formulation. However, there is a subtle indication that an increase in the amount of NFM leads to a slight decrease in moisture content. This observation aligns with the fact that starch is generally more hygroscopic compared to cellulosic materials [57].

The water solubility data is presented in Figure 5b. Despite the relatively large deviation, a discernible trend can be observed. For each set of glycerol content, an increase in NFM content correlates with a decrease in water solubility. This observation is understandable since only PSS is water-soluble, while NFM is not. Additionally, with an increase in glycerol content, water solubility appears to rise. This outcome is expected as glycerol itself is water-soluble. It is also noted that as the glycerol content increases, the water solubility data becomes less scattered.

**Figure 6:**
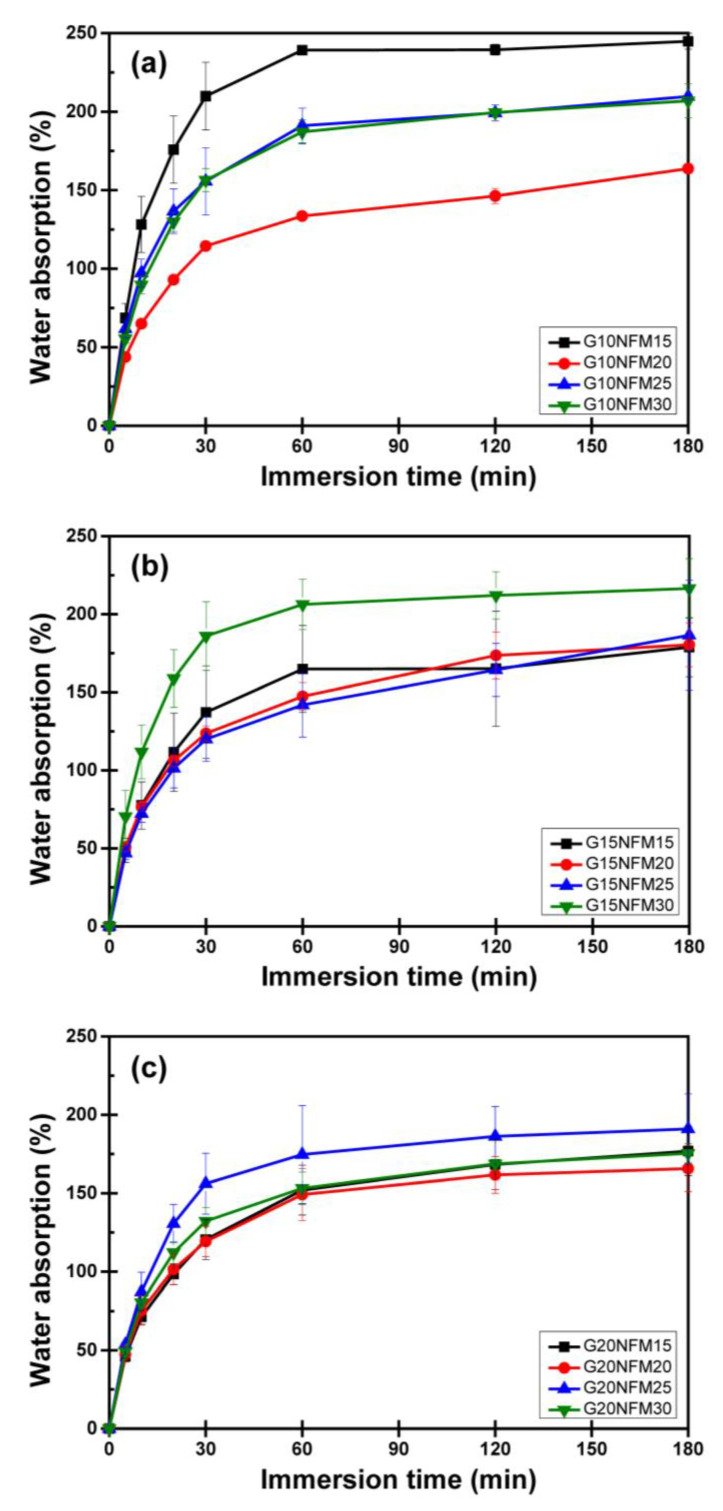
Water absorption of PSS/NFM foams: (**a**) glycerol content 10 wt.%, (**b**) glycerol content 15 wt.% and (**c**) glycerol content 20 wt.%.

Figure 6 illustrates the water-absorption behavior of the foams. All the foams display similar patterns, characterized by a rapid increase in the first 30 min followed by a leveling off. The water absorption values range between 150% and 250%. In the case of the low glycerol content set (G10, Figure 6a), there is a significant variation among different NFM content, and the trend is not readily discernible. However, as the glycerol content increases (Figure 6b,c), the differences between the NFM content sets become smaller, and all the curves converge towards a water absorption level of approximately 150%. This phenomenon is likely related to the changes in the internal structure mentioned earlier, particularly the development of a more uniform void size. The water absorption of PSS/NFM composite foams is about half that of other starch foams [58,59,60].

### 3.6. Soil Burial Test

**Figure 7:**
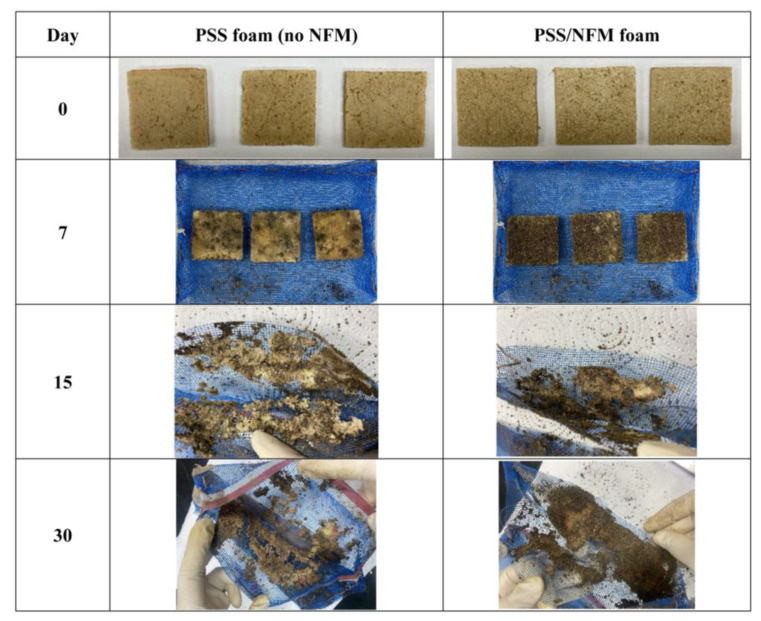
Photographs of some PSS composite specimens before and after being buried in the soil for different periods of time.

**Figure 8:**
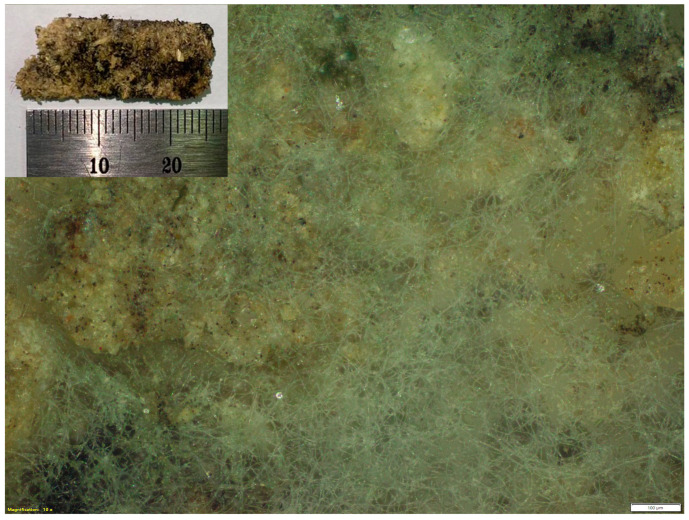
Optical microscope image of G15NFM15 composite foam buried in soil for 7 days (scale bar = 100 µm). Inset is the optical image of the recovered specimen.

Figure 7 displays photographs of some selected PSS and PSS/NFM composite foams before and after the burial test. All foams clearly deteriorated in the burial test within a relatively short period of time. After 7 days, the foams displayed dark stains on the surface but still maintained their original shape. After 15 days, all foams were broken into small pieces and after 30 days completely disintegrated into small particles. Presumably, these tiny particles are cellulosic NFM which is known to degrade at a much slower rate than starch [61]. Further investigation of the dark stains and nearby areas on the surface of the foam using a microscope, as shown in Figure 8, revealed that the area was covered with filamentous materials known as the hyphae of fungi. These findings strongly suggest that the material exhibits biodegradability, sharing a similar mechanism with molds and fungi, which are renowned for their critical roles in the natural environment and their ability to effectively biodegrade organic materials.

## 4. Discussion and Potential Applications

The versatility of PSS/NFM composite foams has been demonstrated through the ease of preparation using various formulations. While the overall density and properties of the foams remain relatively consistent across the formulation range, notable variations are observed in their internal structure. Specifically, a lower glycerol content leads to the presence of larger voids in the core region, whereas a higher glycerol and NFM content result in smaller and more uniform voids.

The increase in NFM content contributes to a slight increase in foam density, primarily due to the increased viscosity of the system and the reduced fraction of the deformable starch phase. These factors limit foam expansion during the foaming process, leading to a denser foam structure.

Despite exhibiting low crystallinity based on XRD results, the PSS/NFM foams exhibit a lower moisture content and water absorption compared to other starch-based materials. This can be attributed to the presence of a small crystalline region, facilitated by the amylose fraction, which acts as physical crosslinking points. These crosslinks effectively prevent the dissolution of the starch network, allowing the foams to retain their structure without disintegration even over extended periods of time in water.

The successful production of biodegradable foams using PSS and NFM derived from pineapple field waste showcases the potential of this approach. By utilizing water as a physical blowing agent and glycerol as a plasticizer to enhance foam durability, we have achieved foam materials that are less fragile. Moreover, this concept demonstrates its circular and sustainable nature when compared to non-biodegradable petroleum-based foams. Additionally, compared to starch foams made from edible starch that require dedicated land and resources for cultivation, our proposed foams primarily utilize waste materials. This results in a reduced energy footprint and significantly lower carbon dioxide emissions of at least 40% (due to the cultivation process) [62] throughout the entire production process. While we acknowledge the importance of providing specific quantitative data to support our claims regarding decreased energy footprint and carbon dioxide emissions, we recognize that further investigation and analysis are necessary. In future publications, we plan to conduct a comprehensive assessment to quantify the exact percentage reduction in energy consumption and carbon dioxide emissions achieved by utilizing waste materials instead of edible starch. This will allow us to present a more detailed and accurate evaluation of the environmental advantages offered by our proposed foams.

**Figure 9:**
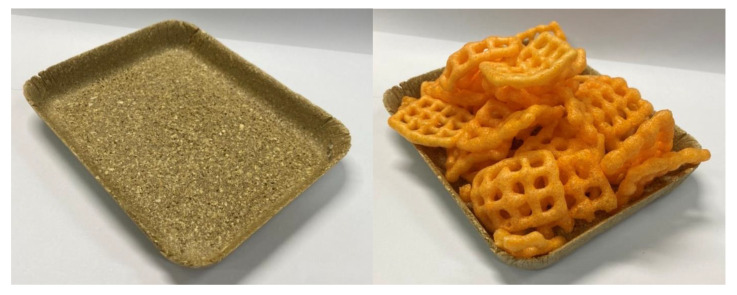
Photographs of packaging tray made from PSS/NFM composite foam.

Furthermore, we have explored potential applications of the PSS/NFM composite, and one notable example is a packaging tray made from our foam material (Figure 9). This tray exhibits acceptable strength and stability while being environmentally friendly and biodegradable. The utilization of our foam material in such applications demonstrates its versatility and suitability for various packaging needs.

In summary, the utilization of PSS and NFM from pineapple waste presents a promising pathway for the production of biodegradable foams. These foams exhibit controlled internal structures, reduced moisture content, and enhanced sustainability. The adoption of waste materials and environmentally friendly manufacturing processes highlights the advantages of this concept over conventional foam materials. Further research and development in this field can contribute to the advancement of sustainable and eco-friendly foam materials.

## 5. Conclusions

This study successfully developed biodegradable foam utilizing pineapple field-waste materials, specifically employing starch extracted from pineapple stem as the matrix and non-fibrous cellulosic materials sourced from pineapple leaf as the filler. The composite foams exhibited controlled internal structures with interconnected cells and relatively thick walls, resulting in densities ranging from 0.43–0.51 g/cm^3^. Notably, the foams demonstrated favorable properties including an equilibrium moisture content of approximately 8–10% and the ability to absorb 150–200% of their own weight without disintegration. Flexural strengths varied between 1.5–4.5 MPa, depending on the filler and glycerol contents. Importantly, biodegradability tests using a soil burial method showed complete disintegration of the foams into particles measuring 1 mm or smaller within a span of 15 days. These findings highlight the promising potential of utilizing waste materials and water as a blowing agent in the production of biodegradable foams. The successful fabrication of an environmentally friendly single-use foam tray further underscores the practicality and versatility of the material. By offering controlled internal structures, improved moisture resistance, and biodegradability, the developed PSS/NFM composite foams present a sustainable alternative to non-biodegradable petroleum-based foams. These outcomes contribute valuable insights to the field of biodegradable foams, paving the way for their broader adoption in various industries and promoting a greener and more sustainable future.

## Figures and Tables

**Table 1:** Codes and composition of PSS/NFM composite foams.

Sample	PSS (wt.%)	Glycerol (wt.%)	NFM (wt.%)
G10NFM15	100	10	15
G10NFM20	100	10	20
G10NFM25	100	10	25
G10NFM30	100	10	30
G15NFM15	100	15	15
G15NFM20	100	15	20
G15NFM25	100	15	25
G15NFM30	100	15	30
G20NFM15	100	20	15
G20NFM20	100	20	20
G20NFM25	100	20	25
G20NFM30	100	20	30

**Table 2:** Density of PSS/NFM foams containing different amounts of NFM and glycerol.

Sample	Density (g/cm^3^)		
NFM (%)	Glycerol 10 wt.%	Glycerol 15 wt.%	Glycerol 20 wt/%
15	0.441 ± 0.007 ^a^	0.427 ± 0.033 ^bc^	0.445 ± 0.022 ^bc^
20	0.453 ± 0.004 ^b^	0.458 ± 0.021 ^c^	0.462 ± 0.019 ^d^
25	0.488 ± 0.021 ^b^	0.454 ± 0.015 ^c^	0.441 ± 0.021 ^b^
30	0.501 ± 0.006 ^b^	0.483 ± 0.011 ^c^	0.512 ± 0.026 ^d^

Note: Different superscript letter for each value indicates statistically significant differences in the means.

## Data Availability

The data presented in this study are available on request from the corresponding author.
